# Best-Corrected Visual Acuity Quantitative Prediction for Cataract Patients: AI-Assisted Clinical Diagnostics Facilitation via the Inverse Problem Algorithm

**DOI:** 10.3390/diagnostics14192126

**Published:** 2024-09-25

**Authors:** Ya-Hui Lin, Chun-Chieh Liang, Ying-Liang Chou, Chih-Sheng Lin, Ke-Lin Chen, Lung-Kwang Pan, Kai-Yuan Cheng, Ching-Hsiu Ke

**Affiliations:** 1 Department of Medical Imaging and Radiological Sciences, Central Taiwan University of Science and Technology, Takun, Taichung 406, Taiwan; ya-hui2@803.org.tw (Y.-H.L.); entchou@gmail.com (Y.-L.C.); lkpan@ctust.edu.tw (L.-K.P.); 2 Department of Clinical Pharmacy, Taichung Armed Forces General Hospital, Taichung 411, Taiwan; 3 Division of Neurosurgery, Department of Surgery, Taichung Armed-Forces General Hospital, Taichung 411, Taiwan; liangchunchieh@gmail.com; 4 Division of Neurosurgery, Department of Surgery, Tri-Service General Hospital, National Defense Medical Center, Taipei 114, Taiwan; 5 Department of Otolaryngology-Head and Neck Surgery, Taichung Armed Forces General Hospital, Taichung 411, Taiwan; 6 Department of Otolaryngology-Head and Neck Surgery, Tri-Service General Hospital, National Defense Medical Center, Taipei 114, Taiwan; 7 Department of Radiology, BenQ Medical Center, Affiliated BenQ Hospital of the Nanjing Medical University, Nanjing 211166, China; chihsheng.lin@benqmedicalcenter.com; 8 Department of Radiology, The First Affiliated Hospital of Ningbo University, Ningbo 315012, China; chenkl2m@me.com; 9 Department of Optometry, Central Taiwan University of Science and Technology, Takun, Taichung 406, Taiwan

**Keywords:** cataract patient, inverse problem algorithm, best-corrected visual acuity, clinical diagnosis, risk factor

## Abstract

**Objective:** This study provided a quantitative prediction of best-corrected visual acuity (BCVA) for cataract patients using the inverse problem algorithm (IPA) technique earlier proposed by the authors. **Methods**: To this end, seven risk factors (age, BMI, MAP, IOP, HbA1c, LDL-C, and gender) were linked by a semi-empirical formula by normalizing each factor into a dimensionless range of −1.0 to +1.0. The adopted inverse problem algorithm (IPA) technique was run via a self-developed program in STATISTICA 7.0, featuring a 29-term nonlinear equation considering seven risk factors, cross-interaction between various pairs of factors, and one constant term [7 + (7 × 6)/2 + 1 = 29]. The IPA neglected quadratic, triple, or quadruple factors′ cross-interactions. This study used a dataset of 632 cataract patients to attain a reliable BCVA prediction with a variance of 0.929. A verification dataset of 160 patients with similar symptoms was used to verify this approach′s feasibility, reaching a good correlation with *R*^2^ = 0.909. **Results**: The verification group′s derived average AT (agreement) (9.12 ± 27.00%) indicated a slight deviation between the theoretical prediction and practical BCVA. The significant factors were age, body mass index (BMI), and intraocular pressure (IOP), whereas mean arterial pressure (MAP), hemoglobin A1c (HbA1c), low-density-lipoprotein cholesterol (LDL-C), and gender insignificantly contributed to BCVA. **Conclusions:** The proposed approach is instrumental in AI-assisted clinical diagnosis, yielding robust BCVA predictions for individual cataract patients based on their biological indices before the ophthalmological examination procedure.

## 1. Introduction

With the aging of the global population and the increase in the prevalence of chronic diseases such as diabetes, the incidence of blindness is on the rise. The top three ocular diseases causing blindness are cataracts, glaucoma, and diabetic retinopathy, according to global and local epidemiologic data [1,2]. A detailed investigation of the major ocular diseases that cause cataracts and their correlated risk factors can help develop effective prevention and treatment strategies to reduce the incidence of blindness, improve the quality of visual health in the lives of patients, and achieve the concept of preventive medicine. Quantitative prediction of best-corrected visual acuity (BCVA) for cataract patients is one of the main goals of this study.

Cataracts, the number one cause of eye blindness in Taiwan, affect optimal corrected visual acuity and significantly reduce the visual clarity of a patient′s life. Usually, it takes about 20 min to detect the optimal clarity of vision in both eyes in a routine ocular examination. Patients with cataracts may receive slit lamp irradiation for lens clarity examination, and the inspection index can offer a solid suggestion for a medical doctor to diagonalize the patients for either administration of a given drug or surgical operation. Nevertheless, many researchers have explored retrospective research for decades and tried to correlate risk factors in cataract patients with the idea that they can propose solid suggestions for preventing the irreversible development of cataracts. In doing so, Singh et al. [3] analyzed the risk factors, which include age, BMI, gender, socioeconomic status, smoking, and diabetes, for cataracts in rural and urban populations. Tang et al. [4] and Hugosson and Ekstrom [5] concluded that age-related cataracts had similar factors in China and Sweden, respectively. Becker et al. [6] emphasized the effects of diabetes on cataract patients. Gaskin et al. [7], Thevi, and Godinho [8] predicted the severity of cataracts by quantifying the risk factors from different perspectives. However, the quantifying indices of risk factors were mostly concluded from retrospective studies, and no further numerical computation was performed to estimate the precise contribution of specific factors. Thus, the quantifying index is similar to qualitative referring.

In contrast, the technique defined as inverse problem algorithm (IPA) in this study was applied to solidify the specific contribution of any factor or cross-interaction among factors. As part of the machine learning technique, IPA involves the application of algorithms to automate decision-making processes using models that have not been manually programmed but have been trained on data [9]. Accordingly, the seven risk factors under study are age, body mass index (BMI), mean arterial pressure (MAP), intraocular pressure (IOP), hemoglobin A1c (HbA1c), low-density-lipoprotein cholesterol LDL-C, and gender; the expectation value, best-corrected visual acuity (BCVA), is the computational result of the IPA algorithm. Accordingly, a 29-item first-order semi-empirical formula was derived to fulfill the installed matrix according to 632 patients′ clinical data. It was then verified by the data of another 160 patients to ensure its reproducibility and coincidence. The details on data recruiting, factor normalization, and further elaboration of the IPA are also discussed.

## 2. Methodology

The IPA technique is a lucrative technique for dealing with large datasets containing various data that might be correlated or irrelevant to each other. Unlike Taguchi optimization or Grey relational analysis in the machine learning field, the former is reputed to optimize a single quality characteristic from multiple factors. In contrast, the latter optimizes multiple quality characteristics from multiple factors altogether. Yet, the IPA minimizes the customized loss function as a computational result.

### 2.1. Inverse Problem Algorithm (IPA)

As a useful technique in artificial intelligence devoted to preventive medicine, the inverse problem algorithm (IPA) is reputed to predict the expectation value from numerous data of multiple factors that might be correlated or irrelevant. Specifically, the biological data of every individual patient need to be recruited and rearranged to fulfill the data format to install into the IPA for computation, and the IPA is defined according to the compromised solution of the inverse matrix, as listed below.

Consider the first-order linear equation y=βx, with the expected value *y* linked to the input value *x* by the sensitivity coefficient β. In this study, *y* = *y* [632 × 1] is the actual BCVA of the ocular patient. Given seven risk factors, cross-interaction between various pairs of factors, and one constant term, the respective nonlinear equation contains 29 terms [7 + (7 × 6)/2 + 1 = 29], taking the following simplified and matrix forms, respectively:

(1) Y=VM

(2) $$\left|\begin{array}{c} y_{1}\\ y_{2}\\ y_{3}\\ \vdots \\ y_{n} \end{array}\right|=\left|\begin{array}{ccccc} v_{11} & v_{12} & . & . & v_{1m}\\ v_{21} & v_{22} & . & . & v_{2m}\\ v_{31} & v_{32} & . & . & v_{3m}\\ \vdots & \vdots & \vdots & \vdots & \vdots \\ v_{n1} & v_{n2} & . & . & v_{nm} \end{array}\right|\left|\begin{array}{c} M_{1}\\ M_{2}\\ M_{3}\\ \vdots \\ M_{m} \end{array}\right|$$

Adopting the standard loss function ∅, we obtain:

(3) $${\emptyset =\left\|VM-Y\right\|}_{2}^{2}\;\;$$

(4) $${𝛻}_{M}\emptyset =2\left(V^{T}\cdot VM-V^{T}Y\right)=0$$

(5) $$V^{T}\cdot VM=V^{T}Y$$

(6) $$M={\left(V^{T}\cdot V\right)}^{-1}\cdot V^{T}\cdot Y$$

In Equations (4)–(6), *V* and *V*^T^ represent the direct and transpose dataset matrices, respectively, considering the seven risk factors under study and available cross-interactions between various pairs of these factors [632 × 29]. Insofar as the standard loss function ∅ is assumed to satisfy the *L′Hospital* rule, its extreme values are derived via Equation (4), which implies a zero value of its total differential ${𝛻}_{M}\emptyset .$ According to the inverse problem solution of Priess et al. [10], the 29-term column matrix of coefficients *M* is obtained using the inverse matrix (*V^T^·V*) via Equations (2)–(6). The calculation procedure for deriving the minimal loss function is obtained via the STATISTICA 7.0 default program. The above computation yields a compromised solution in which further user-oriented customization is possible, as shown in previous studies [11,12].

### 2.2. Semi-Empirical Formula Derivation

The adopted IPA ignores any quadratic (e.g., *v*1^2^, *v*2^2^, *v3*^2^, etc.), triple (*v*1^3^, *v*2^3^, *v*3^3^, *v*1 *× v*2 × *v*3, or *v*1 × *v*2 × *v*4, etc.) or quadruple (*v*1^4^, *v*2^4^, *v*3^4^, *v*1 × *v*2 × *v*3 × *v*4, or *v*1 × *v*2 × *v*3 × *v5*, etc.) factors or their cross-interactions. Instead, it constructs semi-empirical formulas featuring single-factor contributions and cross-interactions of all pairs of single factors. It is inferred that multiple cross-interactions can still contribute to the loss function. Still, this contribution is converged to null to be integrated into the final constant term and treated as the final damping value. Notably, a small constant term is always preferable in IPA to imply a stable approach to ideal loss function with low damping in numerical computation. The details of the computational procedure can be found elsewhere [11,12], with the respective Formula (A1) and its brief explanation provided in Appendix A.

### 2.3. BCVA and Seven Risk Factors

The term “best-corrected visual acuity” (BCVA) defines the optimal eye sharpness measured via an eye chart that can be acquired with the best-fitting corrective lenses. The above eye chart or the Snellen chart displays Sloan letters of varying sizes. In clinical trials, BCVA is a key endpoint for evaluating the effectiveness of treatment. An optometrist can evaluate it after the patient wears the maximum positive spherical aberration at a distance of 6 m from the height of each horizontal row of the visual standard. If the derived BCVA is below 0.8, then the patient might have a high risk of amblyopia and cataracts. When treating eye diseases, e.g., age-related macular degeneration or diabetic retinopathy, doctors verify the treatment efficiency by the BCVA check because it directly reflects how the patient responds to that particular therapy and whether it improves the patient′s vision [13].

This study selected the following seven essential biological indices as risk factors: (1) age; (2) body mass index (BMI, kg/m^2^); (3) mean arterial pressure (MAP, mmHg); (4) intraocular pressure (IOP, mmHg); (5) hemoglobin A1c (HbA1c, %); (6) low-density-lipoprotein cholesterol (LDL-C, mg/dL); and (7) gender. Notably, there is a strong correlation between body mass index (BMI) and human metabolic mechanisms, and BMI is derived as a patient′s weight in kg divided by their squared height in m [14]. MAP is the human artery mean pressure per cardiac cycle, reflecting the level of perfusion to organs; it is derived via systolic blood pressure (SBP) and diastolic blood pressure (DBP) as follows: MAP = (SBP + 2·DBP)/3 [15]. Intraocular pressure (IOP) or eye pressure is the eye′s fluid pressure. The continual production and outflow of fluids maintain this pressure [16]. Glycated hemoglobin, or HbA1c, is a form of hemoglobin (Hb) chemically linked to sugar. Most monosaccharides, including glucose, galactose, and fructose, spontaneously bond with hemoglobin in the bloodstream [HbA1c]. Low-density-lipoprotein cholesterol (LDL-C) is one of the human body′s five fat molecule-transporting lipoprotein groups, which occurs in extracellular water.

The above seven risk factors have various dimensions and need normalization from −1 to +1 to allow the STATISTICA 7.0 program to perform the IPA computation procedure with clinical data input, yielding nondimensional risk factor values. To this end, each risk factor reading X* is normalized as follows:

(7) $$X^{*}=\frac{X-\frac{X_{max}+X_{min}}{2}}{\frac{X_{max}-X_{min}}{2}}\;\;\;\;\;\;\;\;\;\;\;\;\;\;\;\;\;\;\;\;\;$$

where *X*, *X_min_*, and *X_max_* are the respective risk factor′s original, minimal, and maximal readings (*V*_1_–*V*_7_). For example, considering that BMI (*V*_2_) maximal and minimal readings were 43.55 and 13.06 kg/m^2^, respectively, the original BMI values (28.44 and 15.00) of patients No. 30 and 515 were normalized to +0.0093 and −0.8725 via Equation (7), achieving the BMI scale between −1.0 and +1.0.

The seven risk factors′ readings and corresponding BCVA values were obtained for the 632 cataract patients with their eye examinations reported in the Taichung Armed Forces General Hospital, Taiwan, from 1 January 2011 to 31 August 2022. Then, a verification group of 160 patients with similar cataract symptoms was randomly selected from the original group of 792 patients (i.e., 160 = 792–632). The survey authorization (Permit No. B202005075) was issued by the IRB of the Tri-Service General Hospital, Taiwan. The particular results are tabulated in Table 1.

### 2.4. IPA Realization via STATISTICA 7.0

The IPA realization was performed via STATISTICA 7.0 software [17]. It used customized loss functions, treating any cross-interactions and correlations among the selected seven risk factors as nonlinear models/evaluations and user-defined regressions. The input data of 632 patients in the main group were normalized from −1 to +1, explicitly deriving the loss function via the Rosenbrock and quasi-Newton-based converged solutions. The above approach was used instead of Rosenbrock pattern search or Simplex because our earlier research [11,12] proved that minimum loss functions obtained by the latter methods were outside the user-defined demand scope.

**Figure 1:**
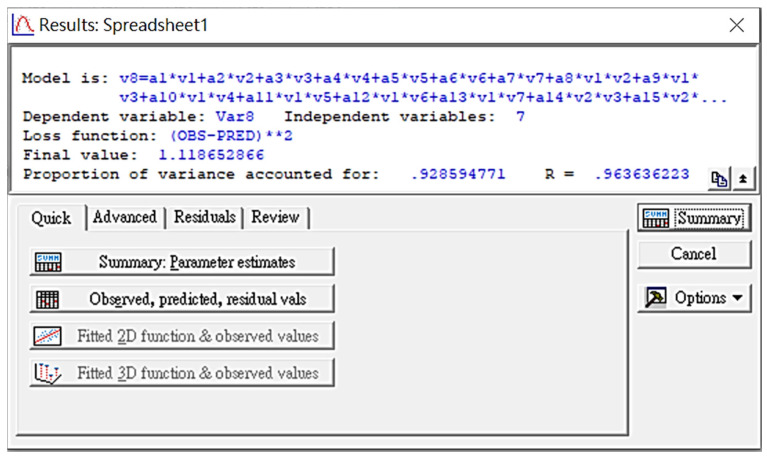
The calculated outcomes from the STATISTICA 7.0 program. The customized loss function equals 0.0 if there is a 100% match between theoretical prediction and practical observation, whereas the derived value is 1.1186 in this study.

Since the expectation values in the above computations were cataract patients′ actual BCVAs, the total number of IPA-incorporated individual data points reached 18,328 (632 × 29 = 18,328). A compromised BCVA column matrix (632 × 1 = 632) was optimized by minimizing the loss function defined as the total fluctuation between predicted and actual BCVA values for 632 cataract patients in the main group. Any links among clinical factors were traced by IPA using a 29-term equation (cf. Equation (A1) in Appendix A). The STATISTICA 7.0 program operation is visualized in Figure 1, implying that one has to follow the proposed option and define the unique loss function to construct the coefficients′ matrix via the IPA technique.

## 3. IPA Results

### 3.1. Numerical Results Obtained

The normalized seven risk factors were computed and are summarized in Table 2. The mean value should be near zero for a normally distributed range [−1.0; +1.0] of a particular risk factor (biological index). The obtained values of BMI (0.26), IOP (0.30), HbA1c (0.25), and LDL-C (0.26) slightly deviate from the standard normal distribution of 632 patients′ data, in contrast to those of age (0.06), MAP (0.22), gender (0.06), and even BCVA (0.08).

### 3.2. Performance Quantification

The IPA results obtained via STATISTICA 7.0 are illustrated in Figure 1. In the ideal case of a 100% match between experimental and predicted BCVA values, a zero customized loss function is expected, while that derived in this study reached 1.118. However, the obtained variance of 0.929 and correlation coefficient of 0.964 implies an excellent fit of IPA-based predictions with the original data matrix values. Table 3 lists the derived coefficients of the 29-term equation (cf. Equation (A1) in Appendix A). Given the above-mentioned [−1; +1] normalization of risk factors, larger coefficients more significantly contribute to the BCVA, with the prevalence of the respective risk factors in the BCVA prediction.

## 4. Discussion

### 4.1. Verification of IPA-Based Predictive Efficiency

**Figure 2:**
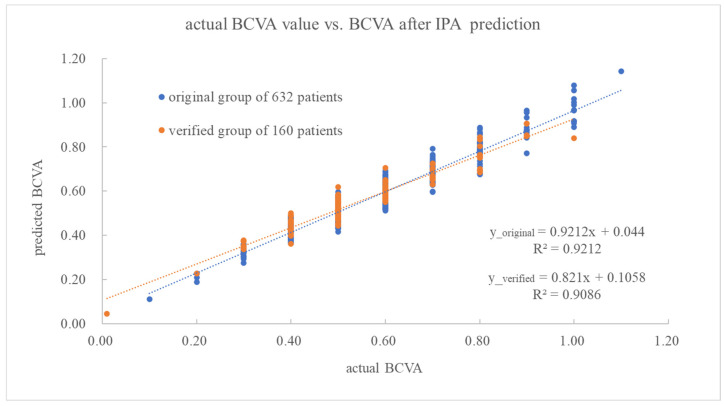
The actual and predicted SYNTAX scores for the original 405 patients and verified 105 patients, according to STATISTICA 7.0-derived linear regression.

To verify the proposed approach′s reliability, the semi-empirical formula (Equation (A1) in Appendix A) with derived coefficients listed in Table 3 was applied to the above-mentioned verification group of 160 patients, whose biological indices were incorporated into the dataset matrix as inputs. The normalized values of each risk factor are summarized in Table 4. Each risk factor′s maximum or minimum value also falls into a similar range as the original group. As seen in Figure 2 and Table 2, the data scatter of the verification and original groups fall into the same range (i.e., the similar slope and r^2^ of two regressed lines). Besides, the linear regression lines of the original and verified group exhibit sensitivities of 0.921 and 0.821, respectively, whereas 1.00 would imply their perfect agreement. Moreover, the correlation coefficients of both lines reach 0.921 and 0.909 (cf. Figure 2), indicating a good fit for IPA-based predictions.

**Figure 3:**
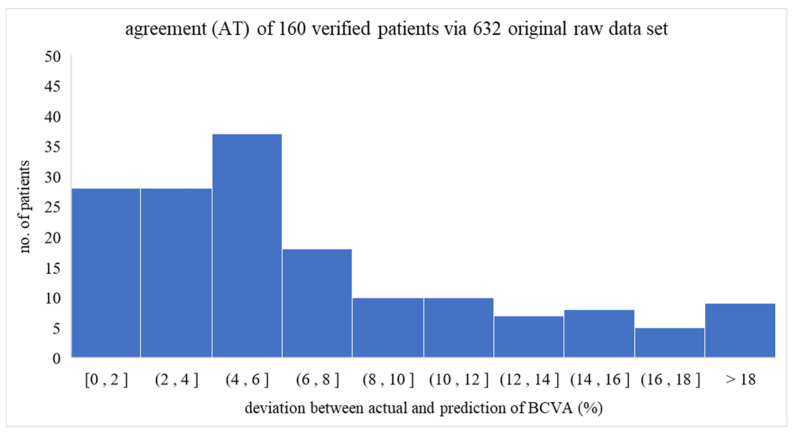
The distribution of 105 individual ATs in this study. As demonstrated, most ATs lie below 6% (i.e., 93 out of 160 below 6%), showing a convincible capability of the program prediction of the BCVA in reality.

The AT parameter (agreement) is derived as follows: AT = [(actual BCVA-predicted BCVA)/actual BCVA × 100%]. In this study, therefore, the average AT_avg_ of 9.12% and standard deviation of 160 ATs of 26.92% confirmed a good fit between the actual and predicted BCVA values [18,19]. The obtained AT distribution of 160 patients depicted in Figure 3 mainly falls below 6.0% (i.e., specifically, 93/160 below 6%), implying a high reliability of BCVA predictions made via the IPA-derived formula.

### 4.2. Significance of Risk Factors in the BCVA Prediction

**Figure 4:**
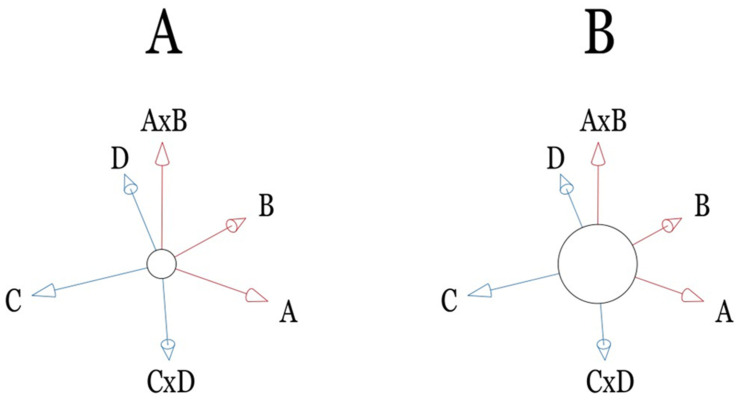
(**A**) A × B has a vertical vector to both A and B and points upward, while the directional vector of C × D points downward; (**B**) Large constant term (implied by the central ball) of the semi-empirical formula can be treated as a stable default value of the expectation of all the individual patients, whereas (**A**) shows a comparatively small constant term (i.e., relatively large oscillation).

Since each adopted risk factor in the proposed IPA approach is normalized to fall into the range from −1.0 to +1.0, its corresponding coefficient in the IPA-derived formula can be interpreted as the significance of the loss function in calculating the BCVA. Each factor significance was derived by its rank as follows: age (2), BMI (5), MAP (25), IOP (7), HbA1c (21), LDL-C (16), and gender (26). Ranking these in decreasing order, age (2), BMI (5), and IOP (7) were identified as significant factors, while LDL-C (16), HbA1c (21), MAP (25), and gender (26) were further considered minor ones. Besides, the cross-interaction between two factors can be interpreted as a new degree of freedom (DoF) for IPA to nullify the loss function: the larger the factor′s coefficient, the faster the IPA convergence, i.e., the faster the calculation results approach zero. As depicted in Figure 4, a large constant can be interpreted as a large nodule at the center point, having a large constant contribution, which is not disturbed by any factor or cross-interaction among factors. Furthermore, a cross-interaction between two factors can create a new DoF that is normal to the original two factors from a mathematical viewpoint. This is similar to a pair of vectors A and B, in which multiplication A × B generates a new vector with a normal direction to the plane of vectors A and B. Notably, the derived constant term in the semi-empirical equation is −0.1345 (ranked 12), which is smaller among all coefficients. Therefore, the loss function can be suppressed only to 0.1187. At the same time, the variance is increased to 0.9285, indicating a good correlation between practical and estimated data (cf. Figure 1) [13].

Accordingly, Age × LDL-C (−0.8183, ranked 1), BMI × LDL-C (−0.5843, ranked 3), Age × MAP (+0.4858, ranked 4), and MAP × LDL-C (+0.2932, ranked 6) are the four most significant cross-interactions among factors. Therefore, it is riskier for aged patients with high LDL-C to have a cataract because it is the number one dominant factor for having serious cataracts in clinical diagnosis. The assumption needs further verification; however, the new interpretation of IPA can help researchers quantify the risk factor′s contribution from different perspectives.

### 4.3. Using the Reduced Number of Risk Factors

**Figure 5:**
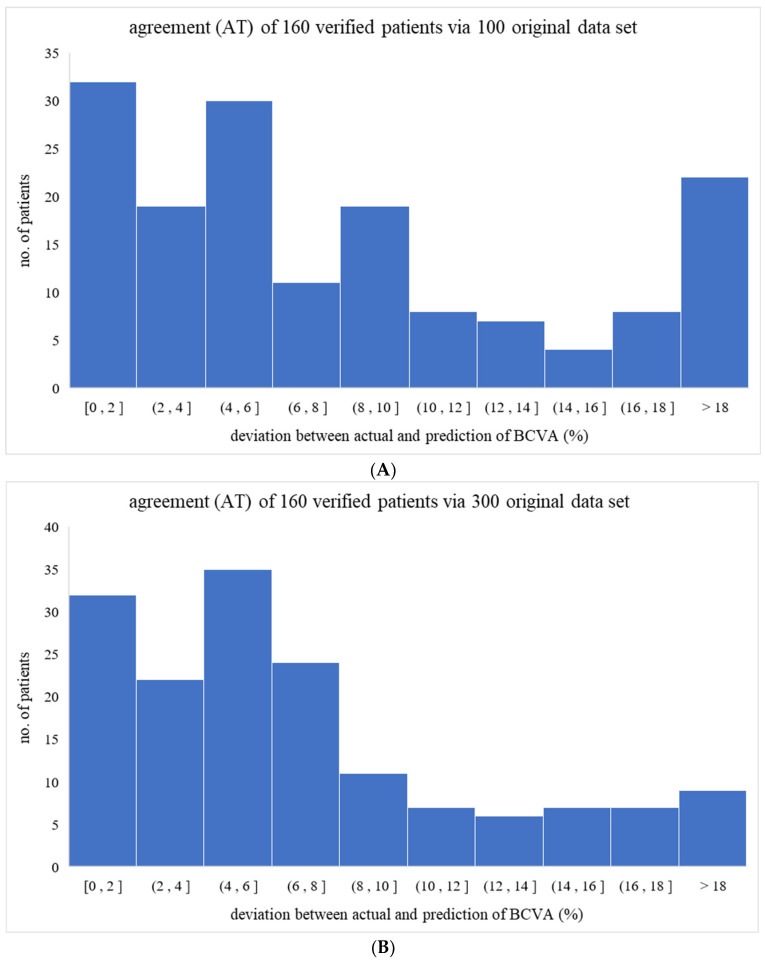
The AT value from the original 9.12 ± 27.00% increased to (**A**) 11.96 ± 43.66% and (**B**) 11.70 ± 60.65% for 100 and 300 original raw data, respectively. In contrast, the AT should approach 0.00 to perfectly match the actual and predicted BCVA in an ideal case.

The proposed IPA prediction was based on analyzing seven biological indices of individual cataract patients. If some data are missing or hard to acquire, the IPA prediction accuracy will remain unchanged due to limited input. This issue was tackled in this study by considering the following two scenarios:

*Scenario I* envisages that the input data are limited to only 100 or 300 patients with similar cataract syndrome, then the IPA is obtained under 100 or 300 data matrices. As shown in Figure 5, the AT values grow from the original 9.12 ± 27.00% to 11.96 ± 43.66% and 11.70 ± 60.65% for 100 and 300 patients, respectively. While the perfect match would result in AT approaching zero, large AT values mean unreliable prediction or wide statistical fluctuations. Quite expected, larger datasets of patients yield more robust predictions than smaller ones.

*Scenario II* implies that one to six of the above seven risk factors (biological indices) are missing or corrupted for some reason. Thus, the IPA prediction must be based on the remaining factors, and the original 29-term semi-empirical formula must be truncated to 22, 16, or even two terms. This scenario was simulated by excluding some risk factors in the decreasing order of their significance, as listed in Table 3: gender (7), MAP (6), LDL-C (5), HbA1c (4), IOP (3), and BMI (2). The truncated formulas featuring six-to-one factors are given in Appendix A as Equations (A2)–(A7). In the further simulations, the biological indices were excluded sequentially, starting from the first one (gender) and ending with the second last (BMI), yielding the respective truncated equation. Accordingly, the final truncated Equation (A7) in Appendix A for *v*2 was defined according to Age (*v*1) only, which most significantly contributed to BCVA prediction.

Table 5 lists predictions with several excluded risk factors. Coefficients in the regressed equations in the fifth column describe a fit of the actual BCVA values and those predicted for the truncated number of various risk factors. A good fit is identified by two criteria [18]: (1) high sensitivity defined by a steep regression curve slope, i.e., large coefficient A in regressed equation *y* = *Ax* + *B*; or (2) high coincidence, i.e., large correlation coefficient *R*^2^ listed in the last column of Table 5. For illustrative purposes, the BCVA values predicted using all seven risk factors are provided in Table 5, showing a gradual accuracy deterioration with each truncation due to the reduced number of risk factors. This finding is also quite expected since all biological indices of patients contribute to deeper insights into their health problems and are instrumental in data processing and analysis by IPA and other artificial-intelligence-aided approaches.

Based on a single risk factor, even the most significant one (namely, age), the IPA computations exhibited a very low correlation coefficient, *R*^2^ = 0.447. Adding BMI as the second significant factor to the list of input variables results in *R*^2^ = 0.499, while adding all remaining six factors yields a good enough value of *R*^2^ = 0.921.

### 4.4. Comparison against Other Findings

Several researchers performed similar studies of cataract risk factors from different perspectives, although their findings are quite controversial. Singh et al. [3] collected data from 1743 cataract patients in India, splitting them into rural and urban subgroups. They reported that age and diabetes were the dominant risk factors for rural patients, whereas age, BMI, and lower socioeconomic status were the main risk factors for urban ones. We included the analytical data from their multivariate analysis to standardize the comparative level. Tang et al. [4] collected cataract patients′ data from Taizhou, China, and revealed that, in addition to age, gender, MAP, and LDL-C, risk factors such as outdoor activity, myopia, and lower income should also be considered. However, a properly quantified evaluation of these additional factors has yet to be performed. Alternatively, Hugosson and Ekstrom [5] analyzed biological indices of 234 cataract patients in Sweden, concluding that age, gender, and myopia were the three most significant risk factors for cataracts, in contrast to pseudo exfoliation, smoking habit, diabetes, hypertension, and ischemic heart disease. Becker et al. [6] processed data from 1152 diabetic cataract patients, reporting that high HbA1c significantly contributed to the cataract syndrome. Gaskin et al. [7] developed a predictive model of risk factors for 8328 cataract patients in the USA and concluded that age, diabetes, and hyperopia were the three dominating risk factors. Thevi and Godinho [8] did a retrospective study and reported that age, MAP, and diabetes heavily impacted cataract syndrome development. Wu et al. [20] evaluated the potential risk factors of posterior capsule opacification after cataract surgery, proving that age, diabetes, myopia immune disease, and lens nucleus hardness were the most significant risk factors. Finally, Chua et al. [21] explored the prevalence, risk factors, and impact of undiagnosed visually significant cataract cases in Singapore, claiming that, besides age and diabetes, factors of ethnicity (Asian, Caucasian, etc.) and educational attainment were also significant.

In contrast, this study ranked the seven risk factors of cataract patients in decreasing order of significance, as follows: age, BMI, IOP, LDL-C, HbA1c, MAP, and gender. Among these, age, BMI, and IOP are significant, whereas LDL-C, HbA1c, MAP, and gender are minor factors. The significance of age is common knowledge that has not been disputed by any research report. The coefficient of age is −0.6798 (ranked 2) according to IPA in this study (cf. Table 3). The BMI significance (ranked 5, −0.3452) has been confirmed by Singh et al. [3]. However, the IOP significance (ranked 7, −0.2852 in this paper) is a unique finding requiring independent experts′ further confirmation based on larger cataract patient datasets. Furthermore, cross-interaction among cataract risk factors can be interpreted as a strong contribution to multivariable analysis. The strongest three cross-interactions are the following: (1) age × LDL-C (ranked 1, −0.8138); (2) BMI × LDL-C (ranked 3, −0.5843); and (3) age × MAP (ranked 4, 0.4858). Thus, the above risk factors are extremely significant for older adults with low MAP or high BMI and LDL-C altogether.

### 4.5. The Proposed IPA Technique Implementation in AI-Aided Diagnostics

**Figure 6:**
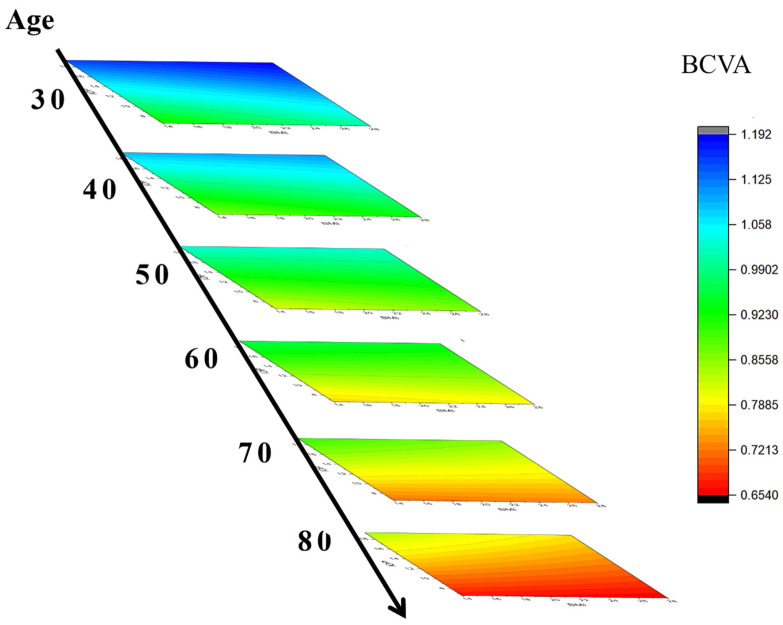
The major three factors, v1 (age, 30–80 yr), v2 (BMI, 13.97−28.01 kg/m^2^), and v3, (IOP, 7–19 mmHg), were preset as the Z- X-, and Y-axes, respectively, in this study. The BCVA is low (<0.80, with the potential risk of cataracts) at the age exceeding 50 and becomes severe for older patients (>60). A high BMI or IOP is beneficial for maintaining high BCVA.

To further explore the IPA technique′s applicability in artificial intelligence (AI)-aided diagnostics, the BCVA predictions made using the above seven biological indices were visualized as a color ladder diagram in Figure 6. Upon normalization, four out of seven biological indices (risk factors) were adjusted to zero values (i.e., 0.0 after normalization, cf. Table 2) to simplify the IPA-based computations for the cataract patient group described in Table 2. Accordingly, the original 29-term equation (cf. Equation (A1) in Appendix A) was reduced to the following 7-term one:

(8) $$\begin{array}{ll} {v8}^{*}=a1\times v1+ & a2\times v2+a3\times v3+a8\times v1\times v2+a9\times v1\times v3\\ & +a14\times v2\times v3+a29 \end{array}$$

A Cartesian coordinate system with three major factors, *v*1 (age, 30–80 yr), *v*2 (BMI, 13.97–28.01 kg/m^2^), and *v*3 (IOP, 7–19 mmHg) assigned along the Z- X-, and Y-axes, respectively, was constructed (cf. Table 3). Low BCVA values (<0.80) indicate a potential risk of cataracts at an age exceeding 50 years, becoming more urgent for older patients over 60 years. Either high BMI or IOP contributes to high BCVA values. In a nutshell, ophthalmologists can assess the BCVA via the truncated semi-empirical formula containing only three major risk factors or obtain a more realistic prediction using all seven risk factors, as proposed in this study.

### 4.6. Challenges of AI-Based Decisions in Preventive Medicine

AI-based decisions in preventive medicine should be double-checked or manually corrected to avoid serious, even lethal incidents. Any set of symptoms may signify several diseases and thus have several solutions. In this case, an AI-based solution can be treated as the one proposed by a human doctor (i.e., it has a bias similar to the human factor). A medical Concilium is a common practice, so the final solution may require enriching the available dataset with additional results from various, more in-depth analyses. Another aspect is exaggerated human trust in new technologies, which force some patients or even unskilled medical interns to seek a diagnosis of serious dysfunction symptoms via AI-based chats (GPT-4, etc.) instead of using professional medical diagnostics. This trend became most pronounced in 2024, when the new version of GPT-4 efficiently solved Ph.D.-level problems, outperforming some human experts. This example proves that the bias from AI systems can be reduced by their continuous upgrading and training since they are only as good as the data they are trained with. Besides, the specific discrepancy between AI-predicted and clinical data must be analyzed to compensate for any potential risk of systematic error and construct a more robust model [22].

Lack of explainability (i.e., no coherent explanation) of AI-detected mechanisms and proposed solutions is typical for any numerical algorithm, in which parameter normalization eclipses their physical meaning. This deficiency applies to the ranking of factors in this study. As seen in Table 6, two factors (HbA1c and LDL-C) were ranked as No.4 and No.5, although they were expected to be among the top three factors since they are related to patient eyesight more directly than others. While other researchers [3,7,20,21] ranked HbA1c as the second most significant factor (see Table 6), LDL-C was either ignored or ranked fourth in [4]. Without coherent explanation, the precedent rule confirms our AI-based ranking of the LDL-C factor. Still, further extension of the cataract patient database is required to refine the HbA1c ranking, which is envisaged in the follow-up study.

The AI trustworthiness issues have been widely discussed for architecture, engineering, and construction solutions [23,24,25], being coupled with challenging ethical aspects in military and medical applications, with human lives at risk. However, the proposed AT-based diagnosis in clinical medicine is only a handy tool prototype, for which further training and refinement via neuron networks is envisaged before its wider implementation. At this research stage, medical doctors must do a further check to verify the patient′s syndrome. The inferred suggestion from an AI algorithm may seemingly contradict clinical common sense, but the fact that it provides a reliable suggestion without any bias from a medical doctor may provide a new suggestion from a different viewpoint for cataract patients with a particular combination of up to seven risk factors.

## 5. Conclusions

Based on the earlier developments of the authors, the IPA-based technique was proposed and tested as an artificial-intelligence-aided clinical diagnostics tool for the quantitative prediction of the best-corrected visual acuity (BCVA) of cataract patients. To this end, a 29-term semi-empirical formula was derived for seven biological indices (age, BMI, MAP, IOP, HbA1c, LDL-C, and gender) treated as the main risk factors. A high variance (0.929) of the actual and predicted BCVA values obtained for the dataset of 632 cataract patients proved the high accuracy of the derived IPA-based solution realized via the STATISTICA 7.0 program, which was further verified in an additional (verification) group of 160 patients with similar symptoms, yielding a correlation coefficient of 0.909. Although a slight deviation between predicted and actual BCVA values was observed and quantitatively assessed via the verification group average AT (AT_avg_ = 9.12 ± 27.00%), the proposed approach is instrumental in clinical diagnostics facilitation. Patients can obtain realistic BCVA predictions based on their individual biological indices before undergoing further ophthalmological examination.

## Figures and Tables

**Table 1:** Raw readings of seven biological indices and actual BCVA values of 632 patients with cataract syndrome having the eye examination, as reported in the Taichung Armed Forces General Hospital, Taiwan, from 1 January 2011, to 31 August 2022.

Risk Factor (Biological Index)	Range		Derived Data		
	Case No./Max.	Case No./Min.	Mean	Median	St. Dev
Age (yr)	#127/103	#20/50	78	77	10.83
BMI (kg/m^2^)	#12/43.55	#276/13.06	24.39	24.03	3.98
MAP (mmHg)	#390/161	#284/57	97.24	95.17	15.43
IOP (mmHg)	#624/35	#171/6	16.13	16	3.66
HbAlc (%)	# 348/15.7	#411/1.5	6.80	6.40	1.88
LDL-C (mg/dL)	#10/234	#219/19	98.69	93	34.96
Gender	#1/+1 (male)	#2/−1 (female)	0	−1	1
BCVA	#239 /1.1	#129/0.1	0.56	0.50	0.14

**Table 2:** Normalized values of seven biological indices and actual BCVA values. Normal distributions of biological indices yield their near-zero mean values.

Risk Factor (Biological Index)	Range		Derived Data		
	Case No./Max.	Case No./Min.	Mean	Median	St. Dev
Age (yr)	#127/+1	#20/−1	0.06	0.02	0.41
BMI (kg/m^2^)	#12/+1	#276/−1	−0.26	−0.28	0.26
MAP (mmHg)	#390/+1	#284/−1	−0.22	−0.26	0.30
IOP (mmHg)	#624/+1	#171/−1	−0.30	−0.31	0.25
HbAlc (%)	#348/+1	#411/−1	−0.25	−0.31	0.26
LDL-C (mg/dL)	#10/+1	#219/−1	−0.26	−0.31	0.33
Gender	#1/+1 (male)	#2/−1 (female)	−0.06	−1.00	1.00
BCVA	#239/+1	#129/−1	−0.08	−0.20	0.28

**Table 3:** Best-fitting coefficients of the 29-term formula (cf. Equation (A1)) computed via the proposed IPA run by the STATISTICA 7.0. Large coefficients indicate more significant contributions of the respective factors to the BCVA prediction.

Risk Factors and Their Cross-Interactions	Factor Abbreviation	Coefficient	Normalized Results	
			Value	Rank
Age	A	*a*_1_	−0.6798	2
BMI	B	*a*_2_	−0.3452	5
MAP	C	*a*_3_	0.0780	25
TOP	D	*a*_4_	−0.2852	7
HbAlc	E	*a*_5_	0.1000	21
LDL-C	F	*a*_6_	0.1231	16
Gender	G	*a*_7_	−0.0419	26
Age × BMI	A × B	*a*_8_	−0.1161	19
Age × MAP	A × C	*a*_9_	0.4858	4
Age × IOP	A × D	*a*_10_	−0.0813	24
Age × HbAlc	A × E	*a*_11_	−0.2330	8
Age × LDL-C	A × F	*a*_12_	−0.8183	1
Age × Gender	A × G	*a*_13_	−0.0327	27
BMI × MAP	B × C	*a*_14_	0.1538	11
BMI × IOP	B × D	*a*_15_	−0.1281	14
BMI × HbAlc	B × E	*a*_16_	0.1287	13
BMI × LDL-C	B × F	*a*_17_	−0.5843	3
BMI × Gender	B × G	*a*_18_	−0.0281	28
MAP × IOP	C × D	*a*_19_	−0.1087	20
MAP × HbAlc	C × E	*a*_20_	0.1808	9
MAP × LDL-C	C × F	*a*_21_	0.2932	6
MAP × Gender	C × G	*a*_22_	0.0086	29
IOP × HbAlc	D × E	*a*_23_	−0.1223	17
IOP × LDL-C	D × F	*a*_24_	−0.1734	10
IOP × Gender	D × G	*a*_25_	0.1274	15
HbAlc × LDL-C	E × F	*a*_26_	−0.1217	18
HbAlc × Gender	E × G	*a*_27_	−0.0982	22
LDL-C × Gender	F × G	*a*_28_	−0.0972	23
Constant term		*a*_29_	−0.1345	12

**Table 4:** Raw readings of seven biological indices and actual BCVA values of the verification group of 160 patients with symptoms similar to those of the original group.

Risk Factor (Biological Index)	Range		Derived Data		
	Case No./Max.	Case No./Min.	Mean	Median	St. Dev
Age (yr)	#85/104	#35/50	77	75	10.55
BMI (kg/m^2^)	#153/39.45	#65/15.22	24.81	24.69	4.43
MAP (mmHg)	#43/148	#117/54	98.36	98.17	13.82
IOP (mmHg)	#31/50	#7/10	17.33	16	4.54
HbAlc (%)	#65/16.6	#52/4.4	6.95	6.45	1.72
LDL-C (mg/dL)	#101/198	#3/16.00	100.62	99.00	31.13
Gender	#2/+1 (male)	#1/−1 (female)	0	−1	1
BCVA	#16/1.00	0.01	0.55	0.50	0.14

**Table 5:** The best-fitting parameters of the linear regression equations derived for various risk factors by STATISTICA 7.0.

Number of Factors Under Study	Number of Terms in the Regression Equation	Loss Function, ∅	Variance of Regression, s^2^	Linear Regression *y* = A*x* + B	Correlation Coefficient, *R*^2^
7	29 (Equation (A1))	1.1186	0.9285	0.9212*x* + 0.044	0.9212
6	22 (Equation (A2))	5.9649	0.8763	0.8764*x* + 0.0690	0.8764
5	16 (Equation (A3))	8.8742	0.8160	0.8161*x* + 0.1026	0.8161
4	11(Equation (A4))	20.000	0.6854	0.5855*x* + 0.2312	0.5855
3	7 (Equation (A5))	22.3977	0.6367	0.5358*x* + 0.2589	0.5358
2	4 (Equation (A6))	24.1781	0.4988	0.4989*x* + 0.2795	0.4989
1	2 (Equation (A7))	26.7971	0.4445	0.4446*x* + 0.3098	0.4466

**Table 6:** Relevant studies of risk factors influencing cataract development.

Reference	[3]	[3]	[4]	[5]	[6]	[7]	[8]	[20]	[21]	This Work
Region/ Country	Rural India	Urban India	China	Sweden	UK	USA	Malaysia	China	Singapore	Taiwan
Population	1094	649	10,234	234	1152 (diabetes)	8382	12,992	550	925	792
Risk factor:										
1	Age	Age	Age	Age	HbA1c	Age	Age	Age	Age	Age *^3^
2	HbA1c	BMI	Gender	Gender		HbA1c	MAP	HbA1c	HbA1c	BMI
3		Lower SES *^1^	MAP	Myopia		Hyperopia	HbA1c	Myopia	Ethnic group	TOP
4			LDL-C					Immune disease	Educational attainment	LDL-C
5			Outdoor activity					LNH *^2^		HbA1c
6			Myopia							MAP
7			Lower-income							Gender

*1. SES is social-economic status; *2. LNH is lens nucleus hardness; *3. Risk factors are listed in the decreasing order of their significance.

## Data Availability

The original contributions presented in the study are included in the article, further inquiries can be directed to the corresponding authors.

## Appendix A

The mathematical expression of the proposed semi-empirical formula has the following form:

(A1) $$\begin{array}{ll} v8=a1\times v1+ & a2\times v2+a3\times v3+a4\times v4+a5\times v5+a6\times v6+a7\times v7+a8\times v1\times v2\\ & +a9\times v1\times v3+a10\times v1\times v4+a11\times v1\times v5+a12\times v1\times v6+a13\times v1\times v7\\ & +a14\times v2\times v3+a15\times v2\times v4+a16\times v2\times v5+a17\times v2\times v6+a18\times v2\times v7\\ & +a19\times v3\times v4+a20\times v3\times v5+a21\times v3\times v6+a22\times v3\times v7+a23\times v4\times v5\\ & +a24\times v4\times v6+a25\times v4\times v7+a26\times v5\times v6+a27\times v5\times v7+a28\times v6\times v7\\ & +a29 \end{array}$$

The remaining six terms are similarly derived as follows:

(A2) $$\begin{array}{ll} v7=a1\times v1 & +a2\times v2+a3\times v3+a4\times v4+a5\times v5+a6\times v6+a7\times v1\times v2+a8\times v1\times v3\\ & +a9\times v1\times v4+a10\times v1\times v5+a11\times v1\times v6+a12\times v2\times v3+a13\times v2\times v4\\ & +a14\times v2\times v5+a15\times v2\times v6+a16\times v3\times v4+a17\times v3\times v5+a18\times v3\times v6\\ & +a19\times v4\times v5+a20\times v4\times v6+a21v5\times v6+a22 \end{array}$$

(A3) $$\begin{array}{ll} v6=a1\times v1 & +a2\times v2+a3\times v3+a4\times v4+a5\times v5+a6\times v1\times v2+a7\times v1\times v3+a8\times v1\times v4\\ & +a9\times v1\times v5+a10\times v2\times v3+a11\times v2\times v4+a12\times v2\times v5+a13\times v3\times v4\\ & +a14\times v3\times v5+a15\times v4\times v5+a16 \end{array}$$

(A4) $$\begin{array}{ll} v5=a1\times v1 & +a2\times v2+a3\times v3+a4\times v4+a5\times v1\times v2+a6\times v1\times v3+a7\times v1\times v4\\ & +a8\times v2\times v3+a9\times v2\times v4+a10\times v3\times v4+a11 \end{array}$$

(A5) $$\begin{array}{ll} v4=a1\times v1 & +a2\times v2+a3\times v3+a4v1\times v2+a5\times v1\times v3+a6\times v2\times v3\\ & +a7 \end{array}$$

(A6) v3=a1×v1+a2×v2+a3×v1×v2+a4

(A7) v2=a1×v1+a2
